# Socioeconomic and disability consequences of injuries in the Sudan: a community-based survey in Khartoum State

**DOI:** 10.1136/injuryprev-2013-040818

**Published:** 2013-11-13

**Authors:** Sally El Tayeb, Safa Abdalla, Ivar Heuch, Graziella Van den Bergh

**Affiliations:** 1 Centre for International Health, University of Bergen, Overlege Danielsens Hus, Bergen, Norway; 2 School of Medicine, Ahfad University for Women, Omdurman, Sudan; 3 Sudanese Public Health Consultancy Group, UK; 4 Department of Mathematics, University of Bergen, Bergen, Norway; 5 Faculty of Health and Social Sciences, Bergen University College, Bergen, Norway

## Abstract

**Background:**

Fatal and non-fatal injuries are of increasing public health concern globally, particularly in low and middle-income countries. Injuries sustained by individuals also impact society, creating a loss of productivity with serious economic consequences. In Sudan, there is no documentation of the burden of injuries on individuals and society.

**Methods:**

A community-based survey was performed in Khartoum State, using a stratified two-stage cluster sampling technique. Households were selected in each cluster by systematic random sampling. Face-to-face interviews during October and November 2010 were conducted. Fatal injuries occurring during 5 years preceding the survey and non-fatal injuries occurring during 12 months preceding interviews were included.

**Results:**

The total number of individuals included was 5661, residing in 973 households. There were 28 deaths due to injuries out of a total of 129 reported deaths over 5 years. A total of 441 cases of non-fatal injuries occurred during the 12 months preceding the survey. The number of disability days differed significantly between mechanisms of injury. Road traffic crashes and falls caused the longest duration of disability. Men had a higher probability than women of losing a job due to an injury.

**Conclusions:**

This study demonstrates the importance of prioritising prevention of road traffic crashes and falls. The loss of productivity in lower socioeconomic strata highlights the need for social security policies. Further research is needed for estimating the economic cost of injuries in Sudan.

## Introduction

Injuries result in 5.1 million deaths annually, and are responsible for 10% of the global mortality.^1^ Sub-Saharan Africa has an estimated 83.2 injury deaths per 100 000 population.^1^ Fatal injury causes irreversible loss of a productive member of society. Millions suffer the consequences of non-fatal injuries, either through temporary disability or life-long sequels. This is reflected in a considerable number of hospitalisation days and loss of productivity. The annual global cost of road traffic injuries alone is an estimated at US$518 billion.^2^ Low and middle-income countries (LMIC) bear 13% of this cost, which amounts to US$65 billion,^3^ and corresponds to a loss of 1–1.5% of the annual national product in LMICs.^3^

In a previous paper,^4^ we reported incidence and risk factors of injuries in Khartoum State, Sudan. The overall incidence was 82.0/1000 person-years-at-risk, and people with low socioeconomic status were found to be at higher risk.^4^ This is consistent with results from other studies showing that poor communities are most affected by injuries even within high-income countries.^5^ Therefore, LMICs have a higher probability of being affected by injuries, fatal as well as non-fatal.^6^

In Sudan, there are no reliable sources documenting injury death. Data extracted from mortuaries are incomplete, and not all deaths are referred for postmortem examination. Development of a fatal injury surveillance system in hospitals and mortuaries is essential but not enough to overcome the data gaps. There is no study which we know of that has reported the consequences of injuries. Evidence-based knowledge is needed to plan and promote prevention programmes, as well as appropriate healthcare and welfare services. The objective of this paper is to determine socioeconomic consequences of disability caused by injuries in Khartoum State, Sudan.

## Methods

A retrospective cross-sectional survey was conducted in Khartoum State from October 2010 to November 2010. Khartoum State is divided into seven localities and further divided into 1496 popular administrative units (PAU). The PAU is the smallest geographically bordered unit identified as our cluster. The total population of Khartoum State is 5.2 million,^7^ equivalent to 17% of Sudan's population.

Sample size calculation was based on a presumed injury prevalence of 50% with 95% confidence level, 5% absolute precision, design effect of two and an average household size of six.^7–9^ The calculated target sample size was 1006 households. The sample was powered to calculate incidence for specific interest groups defined by age, gender and socioeconomic status.^8^ The sampling technique has been described in detail elsewhere.^4^ Briefly, a stratified two-stage cluster sampling technique with the probability of selection proportionate to size was applied.^10^ The state was divided into two strata, urban and rural. The most recent sampling frame from the Central Bureau of Statistics (CBS) was used.^7^ The borders of the PAU/cluster were defined by the community leader. The PAUs in each stratum were listed and random samples of 40 urban and 10 rural clusters were selected. Clusters containing more than 200 households were divided into smaller segments. All segments were given an equal probability of being chosen by a draw. The segment chosen had all households listed by the team, as no reliable up-to-date household address system was available. The listing was accomplished on the day of data collection to ensure minimal mobility of the population sampled. Two PAUs found with abandoned buildings or no residential structures were replaced by adjacent PAUs in the sampling frame.

In the second stage, 20 households were sampled from each cluster applying random systematic sampling. A household was defined as a group of people who most often belong to the same family, who not only live together but also eat and share the same food source.^8^ The first household was selected by dividing the total number of households listed by 20 to get a sampling interval. Then a number was drawn from the sampling interval to identify the first household. The sampling interval was added to identify the rest until the total sample of 20 households had been chosen.^7^ ^9^

### Data collection

Data were collected using three questionnaires. The first questionnaire was structured, collecting sociodemographic information on all household members and housing characteristics, using variables from Sudan Household Health Survey.^11^ The second questionnaire, administered to injured persons, was developed based on WHO guidelines for surveys on injuries and violence.^8^ The WHO definition of an injury was explained to respondents, with examples of injury mechanisms. A 12-month recall period was used. The survey tool used to explore the nature of injuries and body sites was developed using existing surveys (UNICEF/TASC and the US/NHIS surveys).^12^ ^13^ A matrix was developed to reflect the nature of an injury in the body. This allowed covering different types of injury inflicted on different body parts. The first component of disability was defined as the inability to perform normal daily activities. The second component was hospitalisation days as a disability measure. Formal healthcare was defined in our study as treatment at a governmental or private health facility licensed by the Ministry of Health. Economic coping strategies, such as borrowing money or selling belongings as the result of an injury, were also included in the second questionnaire. The third questionnaire was administered in any case of death reported which was due to an injury in the 5 years preceding the interview, so as to capture the rare event of death. Questionnaires were constructed in English and translated to Arabic by a professional translator, and then back-translated for validation by the principal investigator (PI). The survey method and tools were pretested. As a result, modifications to the tools were made, with additional training of the data collectors.

Twelve trained data collectors administered the questionnaire in Arabic to respondents who were preferably female heads of households or eligible adult household members (>18 years of age). In case of an injury, the injured was also interviewed. If this person was absent or below 18 years, an adult proxy was assigned. A household was considered a non-response if found vacant. People living at construction sites, office buildings, schools and shops were included if they cooked and slept at the location. Three field supervisors reviewed questionnaires daily, checking for completion and consistency. The process was repeated by the PI at an office location.

Once data collection was complete, data were entered into CS-Pro V.4.1 (US Census Bureau), with double data entry performed for verification. Data cleaning and analysis was done in PASW V.18 (SPSS). Injuries with at least one day ‘unable to perform normal daily activities’ were included in the analysis. Principal component analysis was used to calculate a composite household wealth index using variables such as home ownership, dwelling type, number of rooms, water source, type of toilet facility, source of lighting, type of fuel used for cooking and assets owned by the household.^14^ ^15^ Categories of socioeconomic status were defined by quintiles of this index. Disability days were calculated for those who fully recovered by computing the days being ‘unable to perform daily normal activities’. Injuries with less than 30 disability days were considered as minor, and injuries with 30 disability days or more were considered major.

The mean and median were calculated from the loss of days. A generalised linear model based on the negative binomial distribution was applied to study associations between hospitalisation days and disability days as dependent variables, and residence, sex, age, education, socioeconomic status, occupation, mechanism of injury and activity as independent variables. χ^2^ Tests were used to test for differences in frequencies between categories.

## Results

A total of 973 households with a total of 5661 individuals consented to be interviewed. The response rate was 97.3%. The average household size was 5.8 individuals. The overall male to female sex ratio was 0.98 : 1, with 38.7% of the sample below the age of 15 years and 16.0% above the age of 45 years. The sample was representative of the state population for age and sex distribution.

### Mortality

There were 28 deaths due to injury out of a total of 129 reported deaths over 5 years. Of the deaths due to injury, 26 were unintentional, 1 was intentional and 1 was of unknown cause. The causes of death were as follows: 17 were due to traffic crashes, 6 were due to falls, 3 due to drowning, 1 due to a gunshot and 1 from an animal bite. The death toll affected males mostly (n=23). A total of 15 victims were above the age of 45 years. The place of death was the site where the injury occurred in 14 cases, a health facility in 10 cases and at home with 4 cases. The majority of deaths occurred immediately (n=13), 6 occurred within less than 24 h, 8 occurred after 24 h and 1 was of unknown timing.

### Body site and nature of injury

A total of 441 persons reported a non-fatal injury which resulted in 1 day or more loss of normal daily activity. Cross-tabulation of body site and mechanism of injury (table 1) showed that the most affected body sites in falls were the upper and lower limbs. The falls were responsible for 60.0% of fractures and 48.1% of dislocations. About 40% of the spine injuries were due to road traffic crashes.

**Table 1:** Body site and nature of injuries for non-fatal injuries, by injury mechanism, Khartoum State study sample, Sudan, 2010

	Falls	Mechanical injuries	Road traffic crashes	Burns	Poisoning	Others	Total n (100%)
Body site n (%)							
Upper limb	62 (32.1)	45 (23.3)	41 (21.2)	27 (14.0)	8 (4.1)	10 (5.2)	193
Lower limb	34 (26.0)	37 (28.2)	23 (17.6)	13 (9.9)	6 (4.6)	18 (13.7)	131
Face	19 (34.5)	20 (36.4)	9 (16.4)	4 (7.3)	0 (0.0)	3 (5.5)	55
Head	9 (25.7)	16 (45.7)	7 (20.0)	2 (5.7)	0 (0.0)	1 (2.9)	35
Abdomen and pelvis	9 (28.1)	2 (6.3)	10 (31.3)	2 (6.3)	9 (28.1)	0 (0.0)	32
Spine	4 (22.2)	5 (27.8)	7 (38.9)	0 (0.0)	0 (0.0)	2 (11.1)	18
Neck	0 (0.0)	4 (100.0)	0 (0.0)	0 (0.0)	0 (0.0)	0 (0.0)	4
Nature of injury n (%)							
Deep injury	22 (21.0)	47 (44.8)	15 (14.3)	0 (0.0)	0 (0.0)	21 (20.0)	105
Superficial injury	34 (36.6)	39 (41.9)	14 (15.1)	0 (0.0)	0 (0.0)	6 (6.5)	93
Fracture	42 (60.0)	10 (14.3)	18 (25.7)	0 (0.0)	0 (0.0)	0 (0.0)	70
Dislocation	26 (48.1)	14 (25.9)	13 (24.1)	0 (0.0)	0 (0.0)	1 (1.9)	54
Burn	0 (0.0)	1 (2.2)	2 (4.4)	40 (88.9)	0 (0.0)	2 (4.4)	45
Poisoning	0 (0.0)	0 (0.0)	0 (0.0)	0 (0.0)	21 (80.8)	5 (19.2)	26
Sprain/strain	7 (46.7)	5 (33.3)	3 (20.0)	0 (0.0)	0 (0.0)	0 (0.0)	15
Concussion	1 (25.0)	2 (50.0)	1 (25.0)	0 (0.0)	0 (0.0)	0 (0.0)	4
Internal injury	0 (0.0)	0 (0.0)	0 (0.0)	0 (0.0)	0 (0.0)	0 (0.0)	0
Others	5 (23.8)	7 (33.3)	6 (28.6)	0 (0.0)	0 (0.0)	3 (14.3)	21

### Disability

Among the non-fatal injuries, 48 (10.9%) claimed they had a permanent disability. A total of 320 persons with non-fatal injuries claimed they had suffered a physical disability as a consequence of an injury (table 2). Major presentations of disability were limping and inability/difficulty of using a hand/arm.

**Table 2:** Characteristics of acquired physical disability among those injured in the past 12 months preceding the interview, Khartoum State study sample, Sudan, 2010

Physical disability	n	Per cent
Walk with a limp	109	34.1
Inability/difficulty in using hand/arm	97	30.3
Weakness or shortness of breath	10	3.1
Inability to chew food	7	2.2
Loss of hearing/loss of vision	5	1.6
Inability to remember things	2	0.6
Other	90	28.1
Total	320	100.0

Tables 3 and 4 present the mean and median hospitalisation and disability days per injury. The numbers are based on injuries with at least one day reported in the relevant category. Mean hospitalisation days depended significantly on socioeconomic status, mechanism and activity (table 3). Mean disability days differed significantly between categories of education, occupation and mechanism (table 4). Low socioeconomic status was associated with long hospitalisation (mean 44.5 days) and short disability period (mean 15.7 days). Unemployed or retired persons still had a long hospitalisation. Falls had a mean hospitalisation of 14.7 days, followed by traffic crashes with 13.8 days. Road traffic crashes led to the longest period of disability (25.8 days) followed by falls (17.0 days). The most serious injuries in terms of disability days occurred during paid work.

**Table 3:** Mean and median hospitalisation for admitted in-patients and disability days of fully recovered injured persons, by demographic factors, for fully recovered injured persons in Khartoum State study sample, Sudan, 2010

	Accessed formal healthcare (n)	Hospitalisation days for admitted		Total (n)	Unadjusted p Value*	Adjusted p Value*†	Disability days (all persons injured)		Total (n)	Unadjusted p Value*	Adjusted p Value*†	Total injured (n)
		Mean	Median				Mean	Median				
Totals	260	10.5	1.7	92			14.9	6.6	294			441
Residence					0.03	0.20				0.004	0.20	
Urban	216	12.6	1.6	70			16.2	7.0	241			355
Rural	44	3.9	1.8	22			9.2	3.8	53			88
Sex					0.03	0.23				0.008	0.08	
Male	171	12.9	1.6	67			17.3	6.7	177			272
Female	89	4.0	1.7	25			11.4	6.1	117			169
Age groups (yrs)					0.007	0.37				0.04	0.28	
0–15	88	2.6	1.2	22			11.5	4.9	112			168
16–44	121	16.3	1.6	46			16.5	6.8	134			197
45+	51	6.5	3.3	24			18.6	9.3	48			76
Education					0.02	0.24				0.02	0.03	
No education	67	14.4	2.0	21			14.7	5.2	83			112
Primary/khalwa	79	17.2	2.3	31			12.9	5.3	98			146
Secondary	57	3.3	1.7	15			11.3	6.4	58			100
Diploma/university+	57	3.3	1.4	25			22.8	11.0	55			83
Socioeconomic status‡					0.007	0.003				<0.001	0.54	
Lowest	46	44.5	4.0	14			15.7	5.5	68			102
Low	63	3.5	1.5	28			8.9	5.1	79			111
Middle	50	7.6	1.8	17			10.5	7.0	52			74
Higher middle	47	2.8	1.1	16			24.0	6.2	49			79
High	54	4.3	2.3	17			19.4	11.6	46			75
Occupation					0.02	0.33				0.003	0.05	
Elementary occupations	21	31.3	2.5	8			28.1	7.6	20			30
Plant and machine operators	12	2.8	2.8	6			10.0	2.7	9			16
Craft and related trades	19	9.1	2.3	9			26.1	8.5	18			26
Unemployed/retired	22	36.2	5.5	10			17.5	8.1	26			36
Housewife	32	4.9	4.0	7			9.8	3.6	44			59
Clerical/service and sales workers	17	9.3	1.8	7			13.7	6.5	17			24
Managers	2	4.1	2.0	9			30.9	14.0	14			19
Technicians	15	–	–	0			8.5	8.5	2			4
Not applicable	120	3.3	1.3	36			11.8	5.5	144			227

*p Value for homogeneity of means over categories.

†Adjustment for all factors included in tables 3 and 4.

‡Quintiles of wealth index based on factors, such as home ownership, number of rooms and households assests.

Missing data was excluded from the analysis.

**Table 4:** Mean and median hospitalisation days for admitted in-patients and disability days of fully recovered injured persons, by mechanism and activity during injury event, Khartoum State study sample, Sudan, 2010

	Accessed formal healthcare (n)	Hospitalisation days for admitted		Total (n)	Unadjusted p Value*	Adjusted p Value*†	Disability days (all persons injured)		Total (n)	Unadjusted p Value*	Adjusted p Value*†	Total injured (n)
		Mean	Median				Mean	Median				
Totals	260	10.5	1.7	92			14.9	6.6	294			441
Mechanism of injury					0.10	0.03				<0.001	<0.001	
Falls	60	14.7	2.2	28			17.0	7.7	79			129
Road traffic crashes	57	13.8	3.0	30			25.8	9.5	52			72
Violence	6	8.4	1.4	7			15.2	7.3	25			38
Burns	23	3.6	3.3	10			2.8	1.9	26			45
Mechanical injuries	77	3.4	1.0	11			10.6	6.3	59			87
Poisoning	16	0.7	0.7	3			11.5	5.8	33			28
Others	17	1.7	1.0	3			12.3	7.0	20			25
Activity					<0.001	0.03				0.10	0.59	
Paid work	76	19.6	1.9	38			19.2	7.2	65			105
Vital activities	39	10.9	5.5	10			16.9	5.6	47			74
Unpaid work	37	3.9	1.3	12			11.7	5.9	48			65
Sports/leisure/play	71	1.6	1.1	23			14.4	6.5	98			144
Education	8	–	–	0			5.2	5.2	5			9
Other	29	3.3	4.0	9			11.3	5.8	31			44

Missing data was excluded from the analysis.

*p Value for homogeneity of means over categories.

†Adjustment for all factors included in tables 3 and 4.

Table 5 shows the societal burden of non-fatal injuries, as described by the total number of disability days for fully recovered over 12 months prior to the interview, within categories of age, mechanism of injury and socioeconomic status. The crude number of disability days corresponded to a burden of 215 days per year in a population of size 100 000. More disability days were reported by males than females for minor injuries (less than 30 disability days) except in the age group 45+ years. A similar but stronger tendency was observed for major (more than 30 disability days) injuries. For minor and major injuries, the age group of 16–44 years carried the largest number of disability days and contributed to 50.3% of the total. Among major injuries in males, road traffic crashes represented the leading mechanism of injury in terms of the burden to society expressed by disability days. In the same category for females, falls represented the leading cause. Road traffic crashes accounted for 30.5% of all disability days.

**Table 5:** Total number of disability days by minor and major injuries for fully recovered injured persons, Khartoum State study sample, Sudan, 2010

	Minor injuries (1–30 disability days)				Major injuries (>30 disability days)				Total disability days	Per cent
	Males	n	Females	n	Males	n	Females	n		
Age groups (yrs)										
0–15	485	70	239	33	502	8	60	1	1286	29.3
16–44	609	72	414	51	1059	8	127	3	2209	50.3
45+	121	16	274	25	279	3	221	4	895	20.4
Total	1215	158	927	109	1840	19	408	8	4390	100.0
Mechanism										
Road traffic	260	31	132	13	889	7	60	1	1341	30.5
Falls	285	38	308	26	524	7	181	3	1298	29.6
Mechanical injuries	293	37	145	19	150	2	40	1	628	14.3
Violence	88	15	80	8	180	1	32	1	380	8.7
Burn	108	10	166	19	0	0	95	2	369	8.4
Poisoning	42	10	30	14	0	0	0	0	72	1.6
Other	27	12	59	6	37	2	0	0	123	2.8
Total	1103	153	920	105	1780	19	408	8	4211	95.9
Socioeconomic status*										
Lowest	269	36	200	27	534	3	67	2	1070	24.4
Low	353	44	186	32	103	2	61	1	703	16.0
Middle	238	31	179	18	130	3	0	0	547	12.5
Higher middle	162	24	155	15	699	7	160	3	1176	26.8
High	193	24	207	17	374	4	120	2	894	20.4
Total	1215	159	927	109	1840	19	408	8	4390	100.0

*Quintiles of wealth index based on factors, such as home ownership, number of rooms and households assests.

The stratification by socioeconomic status showed that for minor injuries, males with low socioeconomic status had the largest total number of reported disability days (353 days). For major injuries, males in the higher middle socioeconomic stratum had the highest number of disability days (699 days).

### Economic impact

Among those injured who had been employed at the time of the injury, a total of 9.3% lost their jobs as a consequence, 34% of whom were heads of households. The percentage differed significantly between genders, 13.4% of men having lost their jobs compared with 4.2% of the women (table 6). In the lower socioeconomic strata, about 15.8% reported to have lost their jobs, and in the higher socioeconomic strata, about 5.4%. When tabulated by injury severity, 18.8% of those affected by major injuries reported losing their jobs.

**Table 6:** Economic consequences of injuries by sex, injury severity and socioeconomic status, Khartoum State study sample, Sudan, 2010

	Sex			Severity			Socioeconomic status*					
	Males	Females	P Value	Minor injuries	Major injuries	p Value	Low	Lower	Middle	Higher middle	High	p Value†
*All injured*												
Household members lost days of work/school			0.78			0.36						0.23
Yes	40	23		33	5		15	17	13	8	10	
(%)	14.7	13.6		12.4	18.5		14.7	15.3	17.6	10.1	13.3	
Household borrowed money‡			0.45			0.34						<0.001
Yes	39	21		33	6.0		17	24	12	6	1	
(%)	14.3	12.4		12.4	22.2		16.7	21.6	16.2	7.6	1.3	
Household sold belongings§			0.49			0.10						0.04
Yes	15	6		8	3		4	11	4	2	0	
(%)	5.5	3.6		3.0	11.1		3.9	9.9	5.4	2.5	0.0	
Household borrowed and sold			0.72			0.24						
Yes	12	6		6	2		3	10	4	1	0	0.13
(%)	4.4	3.6		2.2	7.4		2.9	9.0	5.4	1.3	0.0	
Total	272	169		27	267		102	111	74	79	75	
Lost jobs among those employed at time of an injury			0.05			0.09						0.08
Yes	16	4		7	3		9	6	2	1	2	
(%)	13.4	4.2		5.2	18.8		15.8	12.0	6.7	2.5	5.4	
Total	119	95		134	16		57	50	30	40	37	

*Quintiles of wealth index based on factors, such as home ownership, number of rooms and households assests.

†p Value for trend.

‡No information whether household borrowed money: two persons.

§No information whether household sold belongings: one person.

## Discussion

In this first scientific report of the consequences of injuries in the Sudan, we cover the fatal injuries, various types of non-fatal injuries and socioeconomic and disability aspects in Khartoum State. We collected data on fatal injuries occurring in the past 5 years, but the results obtained were probably subject to bias. Due to memory decay, a long recall period may result in reporting errors, in particular, for less recent deaths.^16^ ^17^ Yet, death due to an injury represented 22% of all deaths reported compared with 10% in the Sudanese census data of 2008 (CBS, personal communication, 2008). In a global perspective, as reported by the World Report on Traffic Injury, road traffic is responsible for 23% of all deaths caused by injuries, compared with 61% in our study.^18^

A clear finding in our study is that injuries mostly affect lower and upper extremities, in accordance with previous results from LMICs.^19^ ^20^ This is also consistent with our finding that the most common physical disabilities are walking with a limp and difficulty using the hand/arm. Information about the nature of injuries is needed for development of trauma care systems 8 21 and it is important to strengthen general surgery, orthopaedic and post-trauma rehabilitation service training in hospitals. It would be of interest for future research to triangulate survey data and hospital records to obtain more valid and complementary estimates.

The hospitalisation day variable as a measure of impact of injuries is a good indicator of severity. However, a deficiency is that those who have no access to formal healthcare cannot contribute to hospitalisation days, leading to an underestimation. In Sudan, a health reform in 1991 resulted in introduction of user fees in public health facilities.^22^ Access to care depends on the ability to pay, and in LMICs, trauma care is limited and costly.^23^ ^24^ Those injured who cannot afford health services in public or private facilities will be forced to choose cheaper alternatives such as traditional medicine. Eventually, this will determine the outcome and extent of complications.^6^

Males represented 60% of the deaths due to injuries in our study, and a similar gender disparity was shown by the US injury mortality study from 1981 to 2007.^25^ Males also reported a greater number of disability days per injury. Males in Sudan, as elsewhere, need to be targeted in prevention programmes.

The crude disability days computed in this study give an impression of the burden on society, comparing categories defined by demographic variables or injury mechanisms. Similar results were reported from Ghana, with the same leading mechanisms but with a significant difference between urban and rural areas.^26^ Among the injury mechanisms, road traffic crashes and falls produced the highest mean number of hospitalisation and disability days. These mechanisms also represented the leading causes in many other studies from LMICs.^27–29^ In the Khartoum setting, road traffic injuries had the highest impact in terms of productivity days lost. Similar patterns were shown in Egypt where road injuries affected men at their most productive age.^30^ Globally, road traffic injuries are considered a major public health concern, and collaborative efforts, especially in LMICs, have been started.^31^ Sudan should join such initiatives and develop measures adapted to the local context. Injury prevention, including road safety initiatives, have proven to be cost effective.^32^

Paid work as activity puts people at risk of being hospitalised. Special attention needs to be paid to effective planning, implementation and evaluation of preventive measures in the workplace within an occupational health framework. This requires strengthening partnership between the Ministry of Health, Ministry of Labour, labour organisations, employers and employees. Economic consequences and coping strategies found in our study in Khartoum, such as borrowing money and selling belongings, consistently affect households from lower socioeconomic strata, as expected in a country where out-of-pocket payments represent more than 95% of health expenditure.^33^ Our results show that the lowest socioeconomic strata had a lower percentage of borrowing money or selling belongings, probably because goods and social networks were not available. A study from Ghana reported similar findings.^34^ People from low socioeconomic strata lost their jobs more frequently due to injuries, probably because they were more likely to be engaged in manual labour work requiring full physical well-being. Other studies have shown how this exacerbates the gap between rich and poor and leads to the poverty trap.^35^ Further research is needed to estimate socioeconomic costs of injuries at different levels, including amounts spent on treating injuries, costs of productive days lost and health system expenses.

A limitation in our study is the recall bias leading to underestimation of the consequences of minor injuries. The 12-month recall period has been shown to affect the extent of reporting of minor injuries in other studies.^17^ ^36^ In this community-based study, data obtained from respondents through face-to-face interviews were difficult to validate. The findings cannot be generalised beyond Khartoum State. The definition of disability is another challenge in measuring the impact of injuries. This can be illustrated through the example of a person with a fractured arm who is still able to attend school or work. The strength of a community-based survey as compared to facility-based studies is that selection bias is minimised. Only the most severe injuries might reach health facilities, and not everybody has access.

This study provided novel information about the nature of injuries and socioeconomic consequences, and about the potential loss of productivity. Injuries due to road traffic crashes and falls were found to have the highest mean number of hospitalisation and disability days. Evidence-based prevention programmes need to focus on these injury mechanisms. Moreover, it is shown that economic coping strategies in lower socioeconomic strata are most depleting. This underlines the need for a health and social security policy with special emphasis on the poor.

**What is already known on this subject**

- Khartoum State, a highly urbanised area of Sudan, has an injury incidence of about 82.0/1000 person-years.
- In low and middle-income countries, the lowest socioeconomic strata are most burdened by injury.

**What this study adds**

- In Khartoum State, duration of disability after an injury depends on educational and occupational status.
- Falls and road traffic injuries lead to a longer duration of hospitalisation and disability.
- People of low socioeconomic status are more likely to lose a job, borrow money or sell household assets than those of high socioeconomic status.
